# KMT Set7/9 affects genotoxic stress response via the Mdm2 axis

**DOI:** 10.18632/oncotarget.4584

**Published:** 2015-08-01

**Authors:** Larissa Lezina, Vasilisa Aksenova, Olga Fedorova, Daria Malikova, Oleg Shuvalov, Alexey V. Antonov, Dmitri Tentler, Alexander V. Garabadgiu, Gerry Melino, Nikolai A. Barlev

**Affiliations:** ^1^ Gene Expression Laboratory, Institute of Cytology, Saint-Petersburg, 194064, Russia; ^2^ MRC Toxicology Unit, Leicester, LE1 9HN, UK; ^3^ Molecular Pharmacology Laboratory, Saint-Petersburg Institute of Technology, Saint-Petersburg, 190013, Russia

**Keywords:** Set7/9, genotoxic stress, operetta, Mdm2, DNA damage

## Abstract

Genotoxic stress inflicted by anti-cancer drugs causes DNA breaks and genome instability. DNA double strand breaks induced by irradiation or pharmacological inhibition of Topoisomerase II activate ATM (ataxia-telangiectasia-mutated) kinase signalling pathway that in turn triggers cell cycle arrest and DNA repair. ATM-dependent gamma-phosphorylation of histone H2Ax and other histone modifications, including ubiquitnylation, promote exchange of histones and recruitment of DNA damage response (DDR) and repair proteins. Signal transduction pathways, besides DDR itself, also control expression of genes whose products cause cell cycle arrest and/or apoptosis thus ultimately affecting the sensitivity of cells to genotoxic stress. In this study, using a number of experimental approaches we provide evidence that lysine-specific methyltransferase (KMT) Set7/9 affects DDR and DNA repair, at least in part, by regulating the expression of an E3 ubiquitin ligase, Mdm2. Furthermore, we show that Set7/9 physically interacts with Mdm2. Several cancer cell lines with inverse expression of Set7/9 and Mdm2 displayed diminished survival in response to genotoxic stress. These findings are signified by our bioinformatics studies suggesting that the unleashed expression of Mdm2 in cancer patients with diminished expression of Set7/9 is associated with poor survival outcome.

## INTRODUCTION

DNA damage is one of the most dangerous forms of cellular stress. Genotoxic stress may be caused by both extrinsic and intrinsic factors, such as gamma- and UV-irradiation, DNA replication stress, reactive oxygen species and others. Among those double-strand breaks are considered to be the most deleterious to cells. Even a single unrepaired break can cause genomic instability and tumorigenesis [1]. There are two major pathways of DNA repair dealing with this type of lesions: non-homologous end joining (NHEJ) and homologous recombination (HR) [2]. These two mechanisms are initiated by several members of the phosphoinositol-3 kinase family (PI3) members exerted by different protein complexes. The first two (ATM and DNA-PK) are activated in response to double-strand breaks (DSB), whereas ATR is activated in response to stalled replication and transcription forks [3]. The signal transduction cascade triggered by these kinases is transmitted further to several other kinases, including Chek1 and Chek2, which, in turn phosphorylate dozens of proteins that coordinate cell cycle checkpoints and the assembly and functioning of DNA repair mechanisms [4].

One of the hallmarks of DNA damage response (DDR) is the formation of γ-H2Ax foci caused by histone H2Ax phosphorylation by ATM/DNA-PK on serine 139 [5]. These modified histones spread in both directions on the distance of megabases from the site of damage and therefore can be easily visualised by microscopy [6]. Propagation of the γ-H2Ax foci depends on the recruitment of DDR factors like MDC1 and the Mre11/Rad50/Nbs1 (MRN) complex, which can then in turn recruit and activate additional ATM molecules, leading to an amplification of the initial signal [7].

In addition to phosphorylation of histones, DNA damage also elicits multiple chromatin post-translational modifications (PTMs) including acetylation, ubiquitinylation and lysine methylation [8]. For example, acetylation of Lys5 (K5) in γ-H2Ax and multiple lysines in histone H4 by Tip60 histone acetyltransferase (HAT) in response to DDR increases the mobility and dynamics of chromatin, thereby facilitating the clearance of damaged DNA from histones and providing an access of DNA repair enzymes to the sites of damage [9]. Importantly, Tip60 loading onto chromatin occurs in histone H3-K9 methylation (H3-K9me) dependent manner [10, 11]. The latter modification is mediated partly by KMT Suv39 h1, which in turn is regulated by another KMT, Set7/9 [12]. Another lysine-specific covalent modification that competes with acetylation is ubiquitinylation. RNF8/RNF168-dependent ubiquitinylation of histone γH2AX and H2B promotes DDR by augmenting the recruitment of 53BP1 to the sites of DNA damage [13–15]. In response to DNA damage histones also undergo lysine methylation, which is important for recruitment of the DDR proteins. For example, lysine methyltransferase (KMT) MMSET methylates histone H4 on K20 (H4-K20) to recruit 53BP1 via its Tudor domain, which specifically recognises methylated lysines [16]. Furthermore, SET8 (PR-SET7) and Suv4-20 h1/h2 also mono- and di-methylate H4-K20 and are found at the sites of DNA damage leading to transient deacetylation of histones [17].

Set7/9 was initially identified as KMT that specifically deposited mono-methyl mark on lysine 4 of histone H3 (H3-K4me1) *in vitro* [18, 19]. However, we and others showed that the recombinant Set7/9 failed to methylate histones as part of nucleosomes [20–22]. This suggests that Set7/9 either methylates free histones or modifies chromatin modifiers thereby indirectly affecting chromatin remodelling. In line with notion, SET7/9 was shown to methylate a number of transcription factors, including TAF10 [23], estrogen receptor α (ERα) [24], RelA [25], PCAF [26], Stat3 [27], Yap [28], Suv39 h1 [12], AR [29, 30], p53 [31] and E2F1 [32].

Importantly, the two targets of SET7/9-mediated methylation, E2F1 and p53, are the critical regulators of not only cell cycle progression and apoptosis, but also participate in DDR [33–35]. E2F1 controls transcription of the CCNE gene, whose product, cyclin E, forms a complex with cdk2 to promote DNA replication [36]. On the contrary, p53 regulates transcription of p21/Cip gene, whose product blunts the activity of cdk2/cyclinE complex and hence, causes cell cycle arrest in G1/S phase. Upon DNA damage, both p53 and E2F1 are stabilised and activated by phosphorylation and acetylation mediated by Tip60, p300/CBP and PCAF [37, 38]. Subsequently, both p53 and E2F1 participate in DNA damage-induced apoptosis: p53 activates transcription of Bax, Puma, Noxa and several other pro-apoptotic genes, whereas E2F1 switches from activation of cell cycle genes to pro-apoptotic TP73 gene [38–40]. Furthermore, both p53 and E2F1 when phosphorylated by ATM can be found at DNA damage foci, suggesting that they can physically recruit DNA repair proteins [41, 42]. One of the critical regulators for both p53 and E2F1 is an E3 ubiquitin ligase, Mdm2. Interestingly, while Mdm2 attenuates the activity of p53 by targeting it for ubiquitin-dependent proteasomal degradation, the transcriptional activity of E2F1 is blunted by Mdm2 without triggering its degradation [38, 43].

Our recent findings highlighted an important role of Set7/9 in DDR. On the one hand, Set7/9 is required for activation of p53 in response to genotoxic stress [44], but on the other hand, the lack of Set7/9 promotes E2F1-dependent transcription of another tumor suppressor, TP73 [38] [45]. These results prompted us to investigate the role of Set7/9 in DNA damage response more closely. In the present study we report that SET7/9 is involved in DDR via additional mechanism that involves Mdm2. Specifically, by using various approaches we demonstrate that forced attenuation of SET7/9 expression is associated with increased DNA damage sensitivity, which, at least partly, is mediated through the regulation of Mdm2 expression.

## RESULTS

### Down-regulation of Set7/9 augments sensitivity to genotoxic stress by doxorubicin

**Figure 1:**
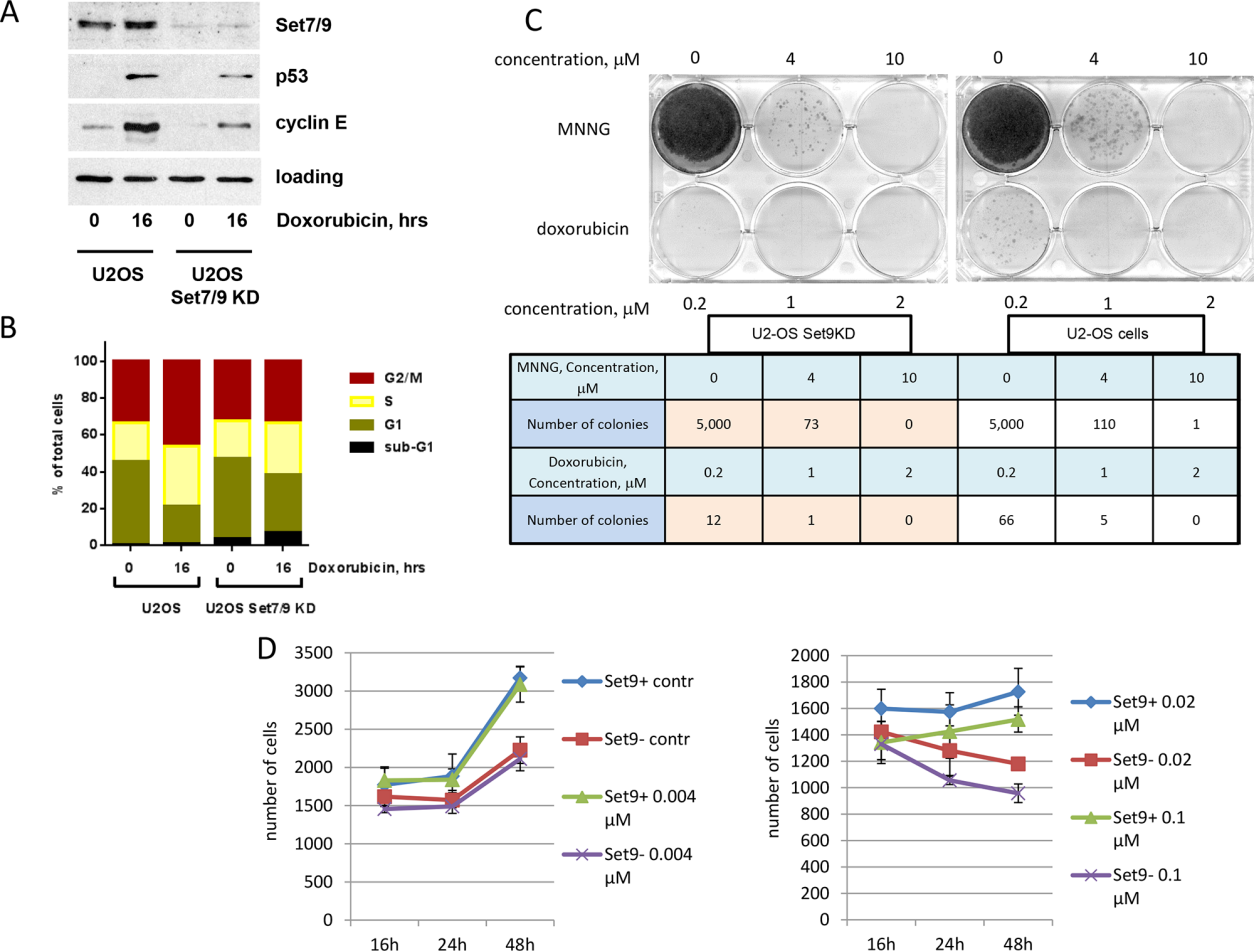
Down-regulation of Set7/9 in U2-OS cells augments sensitivity to genotoxic stress by doxorubicin **A.** U2-OS cells stably expressing control or Set7/9-specific shRNA were treated with 0.5 μM of Doxorubicin for the indicated periods of time and were subsequently analysed by western blotting with the indicated antibodies. **B.** The same cell lines were treated with doxorubicin for the indicated periods of time. The resulting cells were fixed, stained with propidium iodide and analysed for cell cycle distribution. **C.** Clonogenic survival assay of U2-OS control and Set7/9KD cells treated with different concentrations of doxorubicin or MNNG. The numbers of colonies formed in each case are shown in the table below. **D.** U2-OS cells expressing control (Set9+) or Set7/9-specific shRNA (Set9–) were treated with the indicated amounts of doxorubicin for 16, 24 and 48 hours. At each time point the number of cells was determined for both cell lines using an automated microscopy system.

Both p53 and E2F1 transcription factors are activated and stabilised upon DNA damage and regulate DDR by eliciting cell cycle arrest and apoptosis, respectively [33, 38]. Our previous studies have uncovered the role of Set7/9 as an important transcriptional co-regulator for p53 and E2F1 [32, 38, 44, 46, 47]. Based on these findings, we decided to investigate a potential role of Set7/9 in DDR. To this end, we have established a cell line with inducible expression of shRNA against Set7/9 (Set7/9 Knock Down) based on U2-OS (Figure 1A and [45]). Upon induction of shRNA against Set7/9 with doxycycline for three days the level of Set7/9 expression fell down more than five-fold as judged by western blotting.

Upon DNA damage Set7/9KD cells showed attenuated expression of p53 and cyclin E, which is in a good agreement with previously published data [20, 45]. Next, we examined U2-OS Set7/9KD cells along with the parental cells expressing non-specific shRNA for their ability to induce cell cycle arrest and apoptosis in response to genotoxic stress elicited by doxorubicin by FACs (Figure 1B). Due to the over-expression of PP4 phosphatase U2-OS cells elicit G2/M arrest despite the presence of wild-type p53 [48] (Figure 1B). In response to doxorubicin Set7/9KD cells exhibited a more pronounced arrest in G1 and S phases compared to the parental cells. This is due to the compromised E2F1-dependent transcription of the cyclin E gene in these cells [45]. Remarkably, Set7/9KD cells were also more susceptible to apoptosis compared to the control cells (Figure 1B, compare sub-G1 fractions). Taken these results together it was prudent to speculate that the attenuation of Set7/9 may cause DNA damage sensitivity.

We directly explored this possibility using the clonogenic survival assay in which we compared the survival rates of U2-OS Set7/9KD cells with the ones of the parental cells after treatment with different genotoxic compounds (Figure 1C). Prior to this, we examined a range of different concentrations of doxorubicin to establish the efficient dosage of drug to induce cytotoxicity for both cell types (Figure 1D). Low doses of doxorubicin (0.04 and 0.02 μM) did not dramatically affect the growth of both types of cells despite the division time for U2-OS Set7/9KD cells was slightly longer than that of the wild type (Supplementary Figure S1). Since only at 0.1 μM of doxorubicin Set7/9KD and control cells began to show differential sensitivity, we decided to test 0.2 μM concentration of this drug in the clonogenic assay. We also tested the effect of methylnitronitrosoguanidine (MNNG), an alkylating agent that induces DNA mismatch repair [49]. Although Set7/9KD cells showed 1.5 fold increased susceptibility to 4 uM concentration of MNNG compared to the control cells, the effect was not as dramatic as in the case of doxorubicin treatment. At the concentration of 0.2 uM of doxorubicin Set7/9KD cells gave 5.5 times less colonies than the wild-type ones (Figure 1C, bottom row of the table). This result strongly suggests that Set7/9KD cells are prone to DNA damage induced by doxorubicin.

### Set7/9 knockdown impairs DNA damage response and DNA repair

**Figure 2:**
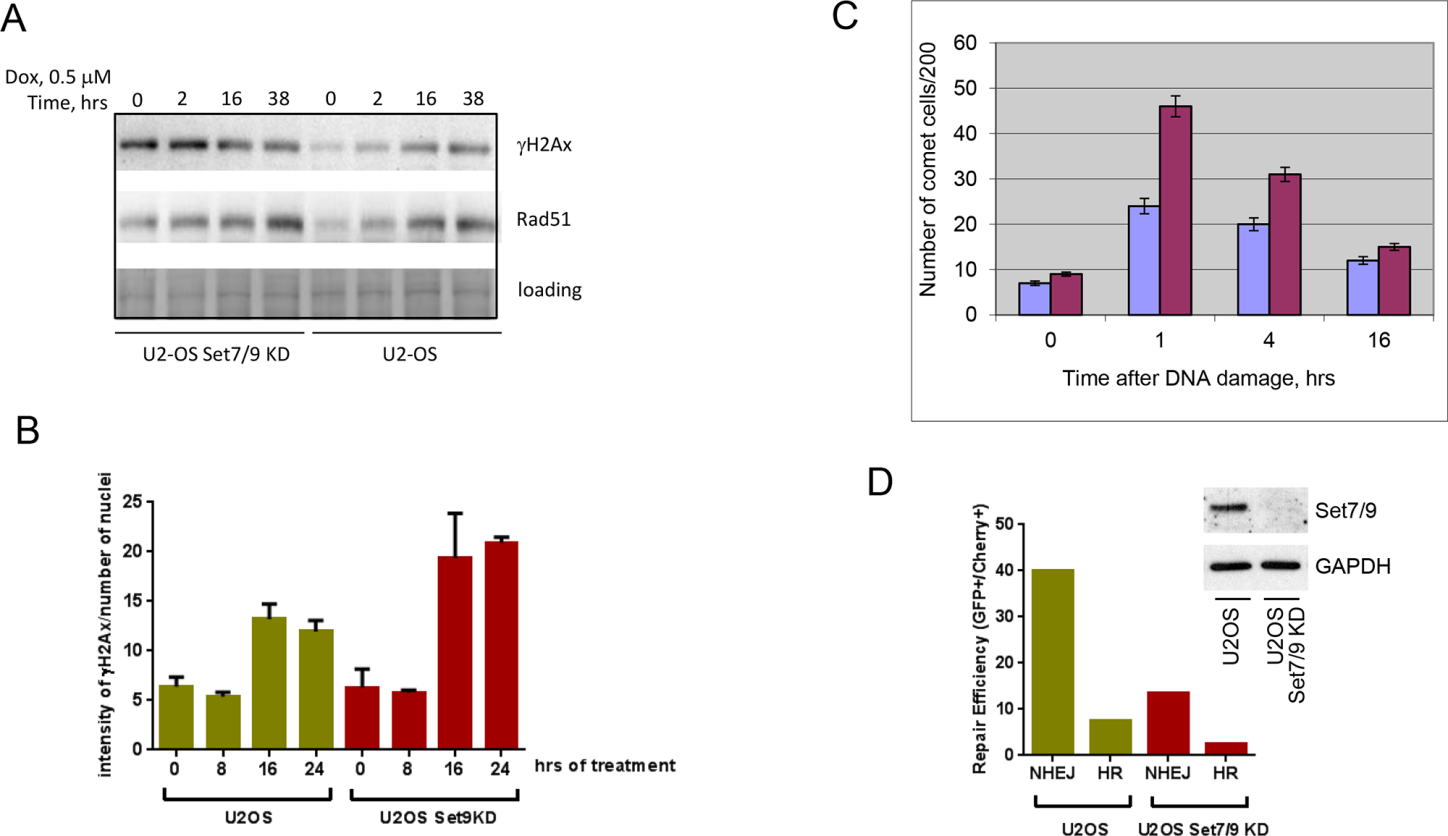
Set7/9 knockdown impairs DNA Damage response and DNA repair **A.** U2-OS cells expressing control (Set9+) or Set7/9-specific shRNA (Set9–) were treated with doxorubicin for the indicated periods of time. Cell extracts for each time point were analyzed by western blotting for expression of γ-H2Ax and Rad51. Coomassie staining (loading) was used for normalization. **B.** U2-OS cells expressing control (Set9+) or Set7/9-specific shRNA (Set9–) were treated with doxorubicin for the indicated periods of time. At each time point the number and intensity of γ-H2Ax foci normalised to the number of cells in the well was determined for both cell lines using an automated microscopy system. Statistical analysis is done by two-way ANOVA. **C.** A representative experiment of the Comet assay in denaturing conditions on U2-OS control or Set7/9KD cells treated with 5 Grey of gamma-irradiation taken at different time points. Experiments were repeated at least three times with similar trend. U2-OS control cells are labeled with blue and U2-OS Set7/9KD cells are denoted with purple, respectively. **D.** DNA repair of double strand breaks in U2-OS control and Set7/9KD cells. The GFP reporter plasmids specific either for HR or NHEJ were digested with I-SceI restriction enzyme before being transfected into U2-OS control and Set7/9KD cells for measuring the efficiency of NHEJ or HR by flow cytometry. The efficiency of Set7/9 knockdown in U2-OS cells before the GFP repair experiment was assessed by western blotting (shown in insert).

The hallmark of DNA damage response in eukaryotic cells is the formation of γ-H2Ax foci. These foci spread on a long distance from the initial site of damage and manifest the activation of ATM/ATR kinases that in turn phosphorylate serine 139 of histone H2Ax [50]. Accordingly, we first assessed levels of γ-H2Ax staining in both U2-OS control and Set7/9KD cells after sustained treatment with doxorubicin (Figure 2A). We found that the level of γ-H2Ax staining in Set7/9KD cells was higher than in the parental cells, especially at early time points (Figure 2A, upper panel). Another well-known marker of DNA damage is Rad51 [51]. Again, the western blot signal of Rad51 was higher in Set7/9KD cells versus control cells (Figure 2A, middle panel), suggesting that DDR is higher in Set7/9KD cells.

The amount of DNA damage is characterized by the number of γ-H2Ax foci and their intensity. To directly assess the effect of doxorubicin treatment on the intensity of γ-H2Ax foci in Set7/9KD and wild-type U2-OS cells we used an automated microscopy “Operetta” to count the intensities of γ-H2Ax foci per nucleus (Figure 2B). Importantly, the level of γ-H2Ax staining obtained by indirect immunofluorescence was also higher in Set7/9KD cells compared to wild-type U2-OS cells.

Because increased sensitivity of Set7/9KD cells to doxorubicin-induced genotoxic stress may be caused by various mechanisms, including the attenuation of ABC transporter activity that controls intracellular levels of the drug [52], we decided to measure directly the levels of DNA repair in U2-OS control and Set7/9KD cells after exposure to 5 Grey of γ-irradiation (Figure 2C). A representative picture of the Comet assay is shown (Figure 2C). As evident from the results of this experiment, consistent with high level of γ-H2Ax staining, more un-repaired DNA remains in Set7/9KD cells in comparison to the control cells.

Doxorubicin is an inhibitor of Topoisomerase II that causes double strand breaks in the DNA. It is known that the double strand DNA breaks repair is largely mediated by two mechanisms: non-homologous end joining (NHEJ) and homologous recombination (HR) [2]. Thus, we sought to determine which mechanism of DNA repair was compromised in U2-OS Set7/9KD cells. To address this question we used a previously described GFP reporter-based system for measuring the efficiency of both types of DNA repair inside cells [53] (Figure 2D). In brief, this system allows monitoring the efficiency of repair of the GFP gene digested with restriction enzymes. The GFP reporter before being repaired by either NHEJ or HR mechanism is inert because of the presence of an inactivating mutation or insertion in the GFP gene, which is subsequently eliminated upon successful repair [53]. As evident from the results shown in Figure 2D both NHEJ and HR repair mechanisms were attenuated in Set7/9KD cells compared to the parental cells. Collectively, these results suggest that the attenuation of Set7/9 expression sensitised cells to DNA damage by affecting both NHEJ and HR DNA repair mechanisms.

### Attenuation of Set7/9 expression increases cellular levels of Mdm2 upon genotoxic stress

To gain insights into molecular mechanisms of DNA damage sensitivity of Set7/9KD cells we performed a genome-wide gene expression analysis of U2-OS and U2-OS Set7/9KD cells before and after treatment with doxorubicin harvested at different time points (Supplementary Figure S2 and data not shown). Since Set7/9 was shown to co-regulate transcription mediated by p53, E2F1, and AR we primarily focused on the known targets of these transcription factors. Another criterion of selection for genes to be studied further was the involvement of their products (directly or indirectly) in DDR and DNA repair. Applying these criteria to the targets of Set7/9 yielded us a gene list shown in Supplementary Figure S2A. Importantly, among these genes was Mdm2, an E3 ubiquitin ligase. Furthermore, Mdm2 is a known target of p53 and the regulator of E2F1 activity, suggesting that Set7/9-dependent DNA damage sensitivity may be mediated by the Mdm2 axis [54].

**Figure 3:**
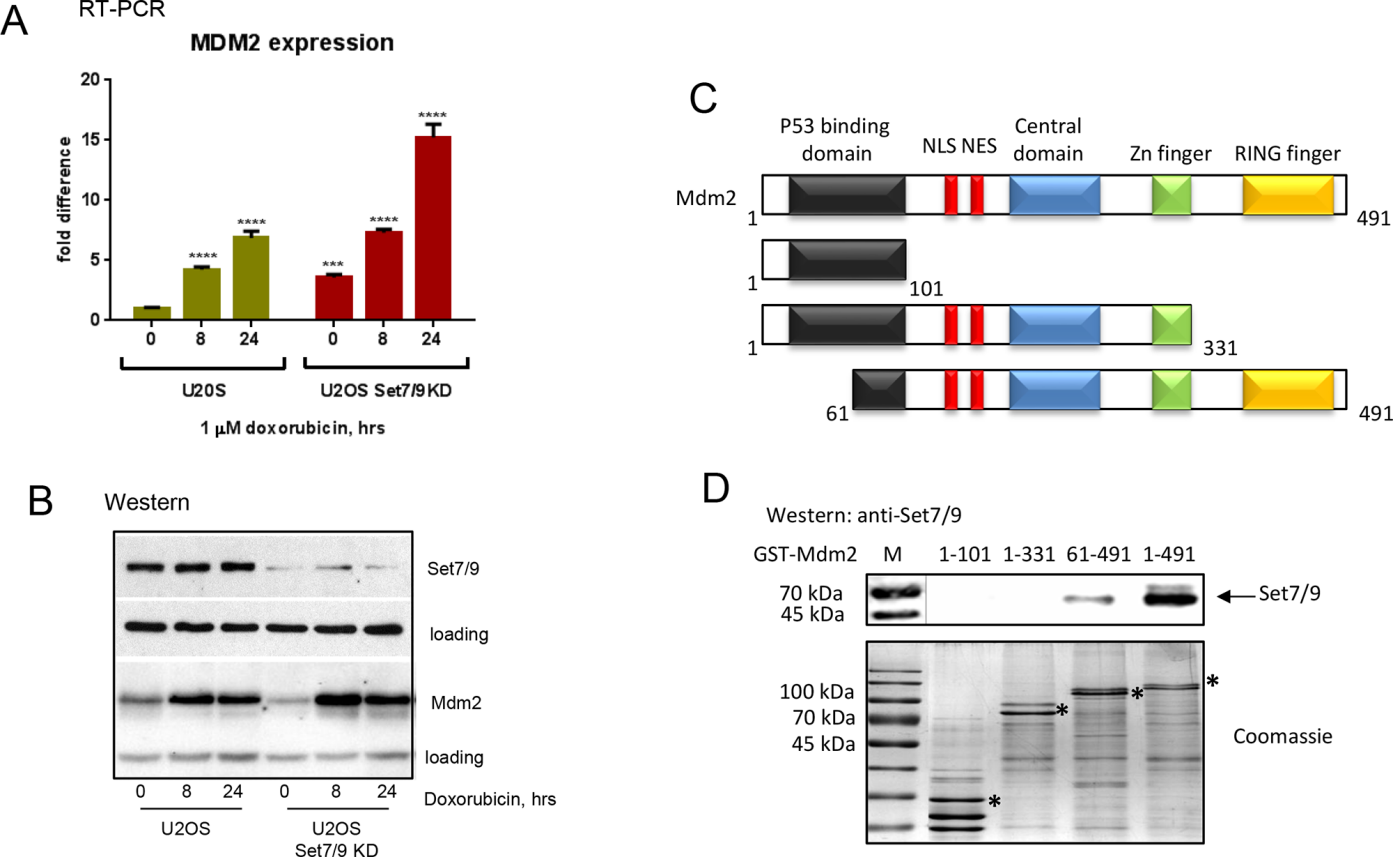
Set7/9 regulates the level of Mdm2 expression upon genotoxic stress **A.** Gene expression analysis of Mdm2 in U2-OS Set7/9 control and Set7/9KD cells by quantitative (q) RT-PCR. Statistical analysis is done by one-way ANOVA. All treated samples are compared with untreated controls of the same cell line. U2OS Set7/9 KD point 0 is also compared with U2OS control (point 0). The number of stars denote: *P < 0.05; **P < 0.01; ***P < 0.001; ****P < 0.0001. **B.** Western blot analysis of Set7/9 and Mdm2 levels in U2-OS control and Set7/9KD cells after treatment with doxorubicin for the indicated times. Note, that the GAPDH signal was used for the normalization of loading in case of Set7/9 and beta-actin in the case of Mdm2. **C.** Schematic representation of Mdm2 functional domains. **D.** Deletion mutants of Mdm2 (dimentions are indicated above) were fused to GST for expression and purification in bacteria on glutathione beads (specific products are labeled with asterics). The same amounts of fusion proteins were used for binding with nuclear extracts prepared from HEK293T cells transfected with Set7/9. The bound material was analyzed for the presence of Set7/9 by western blotting with Set7/9-specific antibody (upper panel).

Next, we validated the results of microarray gene expression experiment by performing (Q)RT-PCR on the Mdm2 gene in U2-OS control and Set7/9KD cells since it has been shown to interact with several well-known regulators of DDR, including NBS1 [55, 56]. Results of (Q)RT-PCR confirmed that Mdm2 was up-regulated in Set7/9KD cells compared to the control cells after DNA damage (Figure 3A). Interestingly, maximal difference in the levels of Mdm2 gene expression between Set7/9KD and control cells was observed at 24 hrs after DNA damage (Figure 3A). Importantly, the difference in the levels of Mdm2 expression was also confirmed by western blotting (Figure 3B). Since Mdm2 has been shown to participate in the regulation of DDR by physical binding with several chromatin-associated factors, including E2F1, PCAF, Tip60 and NBS1 [56], we probed whether Mdm2 also physically associated with Set7/9. To address this, several GST-fusions of truncated Mdm2 mutants (shown in Figure 3C, upper panel) were incubated with cell extracts that contained Set7/9 (Figure 3D). Results of western blotting analysis showed that Set7/9 bound avidly only with the full-length version of Mdm2. However, a much weaker binding was also detected between the Set7/9 protein and the 61-491 fragment of Mdm2. Importantly, protein expression levels of all the Mdm2 mutants were comparable (Figure 3D, upper and lower panels). Apparently, Set7/9 binds both the amino- and carboxyl-termini of Mdm2. Taken these results, we concluded that Set7/9 likely modulates cellular sensitivity to genotoxic stress, at least in part, via Mdm2.

### Mdm2 affects levels of γ-H2Ax upon genotoxic stress

**Figure 4:**
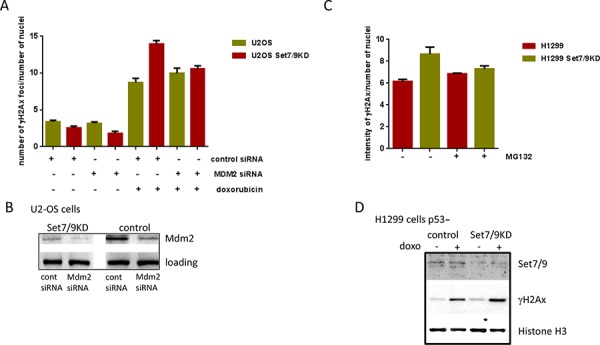
Attenuation of Mdm2 expression levels decreases DDR to doxorubicin **A.** U2-OS Set7/9 control and Set7/9KD cells were treated with control or Mdm2-specific siRNA following the treatment with doxorubicin. The number and intensity of γ-H2Ax foci normalised to the number of cells in the well was determined for both cell lines using an automated microscopy system. **B.** Western blot analysis of U2-OS Set7/9 control and Set7/9KD cells treated with control or Mdm2-specific siRNA. **C.** Proteasome inhibitors decrease DDR in U2-OS Set7/9KD cells. H1299 cells with normal and attenuated expression of Set7/9 were treated with doxorubicin and 1 μM of MG132 or DMSO (control) for 12 hours. The number and intensity of γ-H2Ax foci normalised to the number of cells in the well was determined for both treated and untreated cells using an automated microscopy system. At least three wells were analyzed for each calculation. **D.** Western blot analysis of Set7/9 and γ-H2Ax signals in H1299 control and Set7/9 knockdown cells. Cells were treated for 12 hours or non-treated with doxorubicin and fractionated into soluble nuclear extract and insoluble chromatin. The chromatin fraction was treated with sonication prior to SDS-PAGE, followed by western blotting with Set7/9 and γ-H2Ax antibodies. Histone H3 signal was used for normalization.

To further investigate the role of Mdm2 in Set7/9-mediated DDR, we down-regulated the expression of Mdm2 by specific si-RNA in U2-OS control and Set7/9 KD cells and compared those for the intensity of γ-H2Ax staining after DNA damage induced by doxorubicin (Figure 4A). While there was a minimal difference in γ-H2Ax signals between the control and Mdm2 siRNA samples in the absence of DNA damage, a significant increase in γ-H2Ax staining was detected in U2-OS Set7/9 KD cells versus the control ones after doxorubicin treatment (Figure 4A). Importantly, down-regulation of Mdm2 expression by siRNA in these samples (Figure 4B) negated the difference in γ-H2Ax staining, confirming our hypothesis that Set7/9 operates in DDR, at least in part, via Mdm2.

Next, we asked the question of whether the E3 ubiquitin ligase activity of Mdm2 is important for DDR in Set7/9 KD cells. To address this question we decided to block with MG132 the functional effect of Mdm2 ubiquitinylation activity, i.e. proteasome-mediated degradation of ubiquitinylated proteins [57], and compare the γ-H2Ax signals in MG132-treated and non-treated cells (Figure 4C). To eliminate potential effect of p53 on DNA repair, we used p53-negative lung carcinoma cells, H1299, where the expression of Set7/9 was stably knocked down (Figure 4D). In agreement with our previous results, attenuation of Set7/9 expression in H1299 cells upon sustained treatment with doxorubicin resulted in elevated γ-H2Ax staining compared to Set7/9 control cells (Figure 4C and 4D, middle panel). Notably, the treatment with MG132 decreased the difference in γ-H2Ax signals between H1299 control and Set7/9 KD cells. This result argues that the E3 ubiquitin ligase activity of Mdm2 is important for DDR, although this observation requires additional investigation.

### Attenuated expression of Set7/9 is associated with higher sensitivity to genotoxic stress in lung cancer cell lines

**Figure 5:**
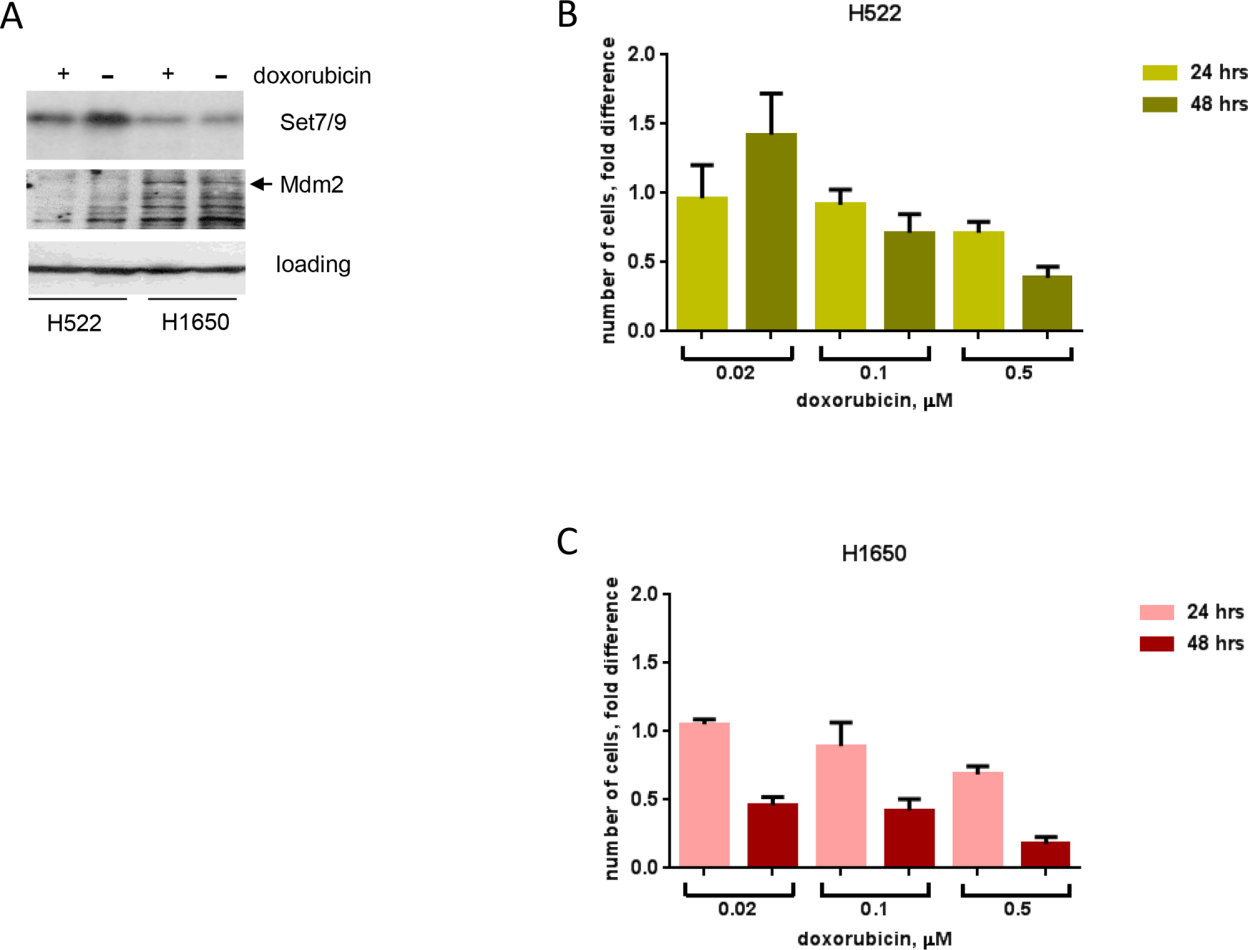
Attenuated expression of Set7/9 in lung cancer cell lines is associated with DNA damage sensitivity **A.** Protein levels of Set7/9 and Mdm2 in H522 and H1650 lung carcinoma cell lines were evaluated by western blotting with the respective antibodies. **B.** Cell survival of H522 after continuous treatment with different doses of doxorubicin (shown in μM) and measured at the indicated time points. **C.** H1650 cells were analysed same as in (A).

We next decided to investigate whether the role of Set7/9 in activation of DDR and cellular cytotoxicity is cell type-dependent. To address this question, we chose two lung cancer cell lines that differentially express Set7/9: H522 cells contain high levels of Set7/9, whereas H1650 cells contain low levels of Set7/9 (Figure 5A, upper panel). Importantly, western blot analysis revealed that H1650 cells expressed higher amounts of Mdm2 compared to H522 cells irrespective of doxorubicin treatment (Figure 5A, middle panel), which is consistent with our results on U2-OS cells that Set7/9 inversely correlates with Mdm2. To assess the role of Set7/9 in DDR, we examined the ability of H522 and H1650 cells to proliferate in response to sustained treatment with doxorubicin. (Figure 5B, 5C). We reasoned that if Set7/9 plays role in DNA damage sensitivity, then H1650 cells should be more prone to doxorubicin compared to H522 as the former express lower levels of Set7/9. Thus, both cell types were continuously treated with increasing amounts of doxorubicin and their growth was monitored by automatic microscopy at 16, 24 and 48 hours (Figure 5B, 5C). To determine the rate of proliferation we arbitrary set the number of cells at 16 h time point as 1 and then plotted the ratios of numbers of cells at 24 and 48 h time points treated with various concentrations of doxorubicin (Figure 5B, 5C). H522 cells (high expression of Set7/9 and low expression of Mdm2) treated with low concentrations of doxorubicin were able to proliferate (Figure 5B, compare the ratios at 24 and 48 hrs at 0.02 μM of doxorubicin). On the contrary, H1650 cells (low expression of Set7/9 and high expression of Mdm2) were extremely sensitive to doxorubicin, as they displayed decreasing ratios of proliferation even at 0.02 μM of doxorubicin (Figure 5C). At higher dose of sustained doxorubicin treatment (0.5 μM) both cell lines showed decreased ratios of proliferation, yet at 48 hours the proliferation ratio was two-fold lower for H1650 cells compared to H522 (compare Figure 5B and 5C, 0.5 μM dose). In support of our hypothesis, H522 cells that express elevated levels of Set7/9 were tolerant even to 0.1 μM dose of doxorubicin, while H1650 cells at this dose underwent cell cycle arrest (Figure 5B and 5C, respectively and data not shown). Thus, we concluded that the correlation between levels of Set7/9 expression and DNA damage resistance does not depend on cell type and may be an important prognostic marker.

### Inverse expression of Set7/9 and Mdm2 correlates with better survival of breast cancer patients

**Figure 6:**
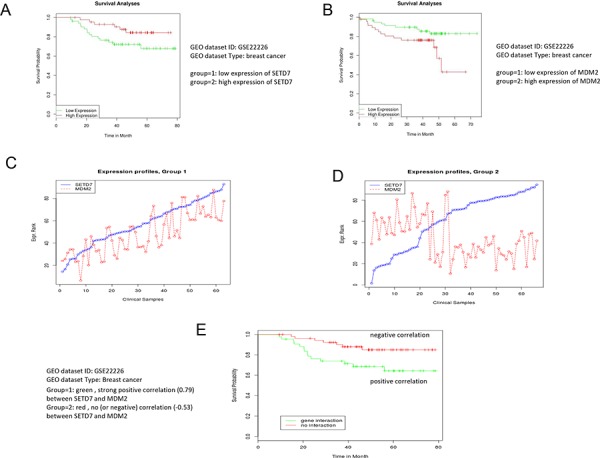
Low expression of Mdm2 correlates with better survival of breast cancer patients The bioinformatics analysis demonstrates that high expression of Set7/9 **A.** and low expression of Mdm2 **B.** in breast cancers correlates with better survival. An illustration of the statistical procedure that divides samples into two groups based on the positive **C.** or negative correlation **D.** between Set7/9 and Mdm2. **E.** Negative correlation between Set7/9 and Mdm2 expression positively affects survival of the breast cancer patients. Correlations for each case are indicated.

To assess the biological significance of our findings, we decided to utilize a bioinformatics approach. Our results suggested that down-regulation of Set7/9 in osteosarcoma U2-OS cells led to defects in DNA repair and hence, enhanced apoptosis (Figure 2D). Since Set7/9KD cells exhibit elevated levels of Mdm2 expression, we questioned whether the product of this gene may negatively affect DNA repair and thus affect the survival of cancer patients. Using the expression data available for breast cancer patients and the algorithm described in [58, 59] we found that high expression of Set7/9 (SetD7) correlated with better survival (Figure 6A). On the contrary, up-regulation of the Mdm2 gene correlated with poor survival outcomes of patients with breast cancers (Figure 6B).

Next, we thought to determine whether a correlation between the expression profiles of Set7/0 and Mdm2 may affect the survival of cancer patients. To achieve this goal we implemented a statistical procedure, which divided the patients into two cohorts. The first one was enriched with positive correlation between the levels of Set7/9 and Mdm2 expression, while all the other patients formed the second cohort (Figure 6C and 6D). To identify statistical differences in survival outcome between the two groups of patients, the R statistical package was used to perform statistical tests and to derive the *P*-value (for the full description of the procedure see [60]). Importantly, we found that the cohort of patients with strong negative correlation between Set7/9 and Mdm2 (−0.53) showed better survival outcome compared with the cohort where Set7/9 and Mdm2 exhibited strong positive correlation (0.59) (Figure 6E). Similar correlation we detected in patients with chronic lymphocytic leukemia (Supplementary Figure S3), suggesting that this observation is not unique to specific type of cancer.

Moreover, we attempted to assess the effect of p53 status on the negative correlation between Set7/9 and Mdm2 expression in breast cancer patients using the METABRIC database [61] (Supplementary Figure S4). To this end, we compared correlation signals between Set7/9 and Mdm2 in two cohorts of patients: with wild type p53 and mutant or no p53 expression (Supplementary Figure S4). Also, we performed the same procedure for two other Set7/9 target genes, HDAC2 and XRCC5 (Supplementary Figure S2A, S2B). Interestingly, the negative correlation between Set7/9 and Mdm2 (−0.17) was detected only in the samples with mutated p53, whereas the correlation for this pair in the samples with wild-type p53 was insignificant (−0.01) (Supplementary Figure S4). However, no statistically significant effect of p53 was observed on correlations between Set7/9 and HDAC2 or XRCC5 (Supplementary Figure S4). Collectively, these results argue that Set7/9 participates in DDR by regulating expression of a number of genes, from which only a portion are transcriptional targets of p53.

## DISCUSSION

In this study we uncovered a novel function of KMT Set7/9 in DDR. Abrogation of Set7/9 expression in U2-OS cells induced their sensitivity to genotoxic stress elicited by anti-cancer drugs (Figure 1C, 1D). This sensitivity was caused by defects in both mechanisms of double strand breaks repair, NHEJ and HR (Figure 2D). Sensitivity of Set7/9KD cells to sustained exposure to genotoxic drugs was accompanied by an increased formation of γ-H2Ax foci, which manifests DDR (Figure 2A, 2B). Microarray gene expression analysis of Set7/9KD and wild-type cells revealed that Set7/9 attenuation resulted in altered expression of a number of genes involved in DNA damage signalling and repair, cell cycle checkpoints, and apoptosis. (Supplementary Figure S2A, S2B). Importantly, the level of p53 expression itself was attenuated in Set7/9KD cells consistent with our previously published results.

One of the principal regulators of p53 activity and stability in the cell is an E3 ubiquitin ligase, Mdm2. The latter is the target of p53 activity itself. Surprisingly, our results suggest that the lack of Set7/9 increases the level of Mdm2 expression (Figure 3A, 3B), despite the attenuation of p53 levels.

To circumvent this discrepancy, we hypothesize that the elevated expression of Mdm2 in Set7/9KD cells depends on other transcriptional factors, e. g. TAp73, which can often substitute p53. Noteworthy, the level of TAp73 expression is elevated in Set7/9KD cells upon DNA damage [45]. Alternatively, the lack of Set7/9 may down-regulate Mdm2-targeting micro-RNAs thus resulting in stabilization of the Mdm2 RNA message. Consequently, Mdm2 interferes with DNA repair by sequestering NBS1 from sites of DNA damage [55, 62]. The functional significance of Mdm2 in DDR is also signified by the fact that down-regulation of the Mdm2 level by siRNA, or its activity by MG132, led to attenuation of the γ-H2Ax staining in Set7/9KD cells upon sustained doxorubicin treatment (Figure 4A, 4C).

It is also important to note that Set7/9 was found to physically interact with Mdm2 (Figure 3D). Apparently, this interaction is complex, as both the amino- and carboxyl termini of Mdm2 are required for the interaction with Set7/9. We assume that in the cells where Set7/9 is expressed at high levels, the latter is able to neutralize the negative effect of Mdm2 on DNA repair.

Alternatively, Set7/9 as an important transcriptional coactivator of several transcription factors may affect DDR via regulation of other genes, besides Mdm2, including long non-coding and micro-RNAs [59, 63, 64]. In this respect, it would be interesting to determine the list of micro-RNAs affected by Set7/9 and whether their gene targets are involved in DDR. This work is under its way.

From the cancer therapy point of view it is important to note that lung carcinoma cell lines H522 and H1650 that differ in the levels of Set7/9 expression also displayed differential sensitivity to genotoxic stress induced by doxorubicin. These data argue that negative correlation between Set7/9 and Mdm2 expression levels may be an important prognostic marker of tumour sensitivity to genotoxic therapies. Our bioinformatics data on breast cancer patients support this notion.

Undoubtedly, more experimental work is required to ascertain the role of Set7/9 in the process of DDR and its effect on cellular cytotoxicity in response to genotoxic stress. These findings may have important ramifications to our understanding of the molecular mechanisms of DDR in tumour cells and help to design novel genotoxic anti-cancer therapies.

## MATERIALS AND METHODS

### Cell lines manipulations

All cells used in this study were purchased from ATCC, if not stated otherwise. Human lung adenocarcinoma cell lines H522 and H1650 were propagated in RPMI medium (Gibco) supplemented with 10% fetal bovine serum (FBS) (Gibco). Human osteosarcoma cell line U2-OS and its derivatives were maintained in DMEM medium (Gibco) supplemented with 10% fetal bovine serum (FBS) (Gibco). Cells were grown at 37°C in a humidified atmosphere with 5% CO_2_. U2-OS cells with Tet-inducible expression of shRNA against Set7/9 and the reference cell line were generated as described in [45]. To manipulate with the expression level of Mdm2 specific siRNA (siRNA-Mdm2) as well as the control one (c-siRNA) were purchased from Ambion Life Technology (Cat#4390824, ID S8630). Typically, 50nM of c-siRNA or siRNA-MDM2 were used for transfection with Lipofectamine 2000 (Life technologies, USA) in Opti-MEM (Gibco, USA). Following two days of incubation to achieve knockdown of Mdm2 expression, cells were treated with doxorubicin as described.

### Cell cycle analysis

For the cell cycle analysis cells were harvested, washed once with PBS, and fixed in 70% ethanol overnight at −20°C. Staining for DNA content was performed with 50 μg/ml propidium iodide (Sigma-Aldrich) and 1 μg/ml RNase A for 30 min. Analysis was performed on a FACS Canto II flow cytometer (BD Biosciences) with Cell Quest Pro software. Cell cycle modelling was performed with Modfit 3.0 software (Verity Software House, Topsham, ME).

### Clonogenic assay

Cells were seeded in amount of 5 × 10^3^ per well and 16 hrs later were treated with 4 and 10 μM MNNG (Sigma-Aldrich) for 1 hr in OPTI-MEM or 0.2, 1, or 2 μM doxorubicin (Sigma-Aldrich) for 2 hrs in DMEM. After treatment cells were washed with PBS and grown in DMEM for 2 weeks. After that time cells were fixed with 10% formalin (Sigma-Aldrich), stained with Giemsa as described [65], and colonies were counted.

### Comet assay

A single-cell suspension with 10^4^ cells per sample was mixed with 1% agarose and placed onto agarose pre-coated slide. Lysis was performed overnight in alkaline conditions as described [66]. Next day slides were rinsed, subjected to electrophoresis, stained with 2.5 mg/ml of propidium iodide (Sigma-Aldrich), and analysed by CometScore 1.5 software. Two hundred cells were manually inspected in each case. Experiments were repeated at least three times showing the same trend.

### DNA repair analysis using reporter constructs for NHEJ and HR

Plasmids carrying NHEJ or HR reporter cassettes containing a GFP gene, as described [53], were linearized by I-SceI (NEB) and used for transfecting cells: 0.5 μg and 2 μg respectively. 0.1 μg of pmCherry vector (Clontech) was co-transfected in both cases as a transfection efficiency control. Transfections were performed using TurboFect transfection reagent (Fermentas). 48 hrs later cells were harvested and analysed by 2-laser FACS.

### RNA isolation and relative quantification RT-PCR

Total RNA was extracted from the cultured cells using TRIzol Reagent (Invitrogen) according to the manufacturer's instructions. For relative quantification RT-PCR analysis of Tip60, MDM2, and GAPDH mRNA, 1 μg of total RNA was reverse-transcribed to cDNA with oligo d(T) using RevertAid H Minus First Strand cDNA Synthesis Kit (Thermo Scientific). cDNA were amplified by real-time PCR on an RotorGene 6000 PCR machine (Qiagene) using SYBR green mix (BioLine). All reactions were run in triplicate. Data were analysed by RotorGene 6000 Series Software. Relative amounts of Tip60 and MDM2 mRNAs were normalized to GAPDH mRNA. Expression of these genes was analysed by RT-PCR using the following primers: (Tip60) F-ttttccccagaatggagccg, R-gtggtgctgacggtattcca; (MDM2) F- tgggcagcttgaagcagttg, R-caggctgccatgtgacctaaga; (GAPDH) F-gggaag gtgaagg tcggagt, R-ttgaggtcaatgaaggggtca.

### Microarray gene expression analysis

Microarray gene expression analysis was performed using Human Gene Expression 4x44K Microarray Kit and Low Input Quick Amp Labeling Kit (Agilent Technologies) according to the manufacturer's instructions. Quality of RNA was tested by 2100 Bioanalyzer (Agilent Technologies). 100 ng of each RNA sample were used for cRNA synthesis and were simultaneously labelled with Cy-3. After purification 1.65 ng of each cRNA sample were hybridised with oligonucleotide probes on microarray slides for 17 hrs. Next day slides were washed and scanned. Data were analysed by GeneSpring GX11.5 software.

### Western blotting

Set7/9, p53, Cyclin E, Tip60, HDAC2, and MDM2 protein levels were quantified by Western blot analysis of whole cell extracts using antibodies against the corresponding proteins. These samples were normalized by blotting with an antibody against GAPDH. The following antibodies were used: anti-Set7/9 (Cell Signaling), anti-p53 (Ab-6, Oncogene), anti-Cyclin E (HE12, Santa Cruz), anti-Tip60 (Millipore), anti-HDAC2 (3F3, Millipore), anti-MDM2 (SMP14, Sigma or Santa Cruz), anti-GAPDH (Abcam).

### Protein-protein interactions

To test the interaction between Mdm2 and Set7/9 *in vitro* 20 μg of recombinant GST-Mdm2 fusion proteins were incubated for 3 hours with 1 mg of nuclear extract prepared from HEK293T cells transfected with Flag-Set7/9 expression construct. Following washes in PBS, bead-bound material was analyzed by western blotting using Set7/9 antibody.

### Automated and confocal immunofluorescence microscopy

The γ-H2Ax intensity levels in U2-OS, H1299 and matching cell lines with knockdown of Set7/9 as well as in H522 and H1650 cell were estimated using automated microscopy (Operetta, Perkin Elmer) at 40X magnification. Typically, for the automated microscopy analysis cell lines were fixed with 4% PFA, permeabilized with 0, 5% Triton X-100 and incubated with anti-γ-H2AX antibodies at 1:500 dilution. To visualize nuclei, cells were stained with Hoechst (3 μg/ml) and were subsequently analysed in the Hoechst channel with exposition time 50 ms; γ-H2Ax foci were analysed in the Alexa 488 channel with exposition time 200 ms. The intensity levels of γ-H2Ax foci were calculated using the following spots parameters: radius < or = 0.95 μm, distance between foci > or = 0.74 μm. Total intensity of γ-H2Ax in foci was normalised to the number of cells in the well.

### Bioinformatics analysis

A detailed description of bioinformatics analyses and algorithms used in this study can be found in [60].

## SUPPLEMENTARY FIGURES


