# Supplementary material for: KMT Set7/9 affects genotoxic stress response via the Mdm2 axis
Source: Oncotarget. 2015 Aug 1;6(28):25843–55. doi: 10.18632/oncotarget.4584 (PMC4694870; doi:10.18632/oncotarget.4584)
Supplement: Supplementary file 1 [file oncotarget-06-25843-s001.pdf]

SUPPLEMENTARY FIGURES

Time	0hrs	8hrs	23hrs	33hrs	46hrs	55hrs
U20S	14	18	27	45	68	101
U2OS Set7/9-knockdown	16	17	28	45	52	73

total cell count x10^4/500µl

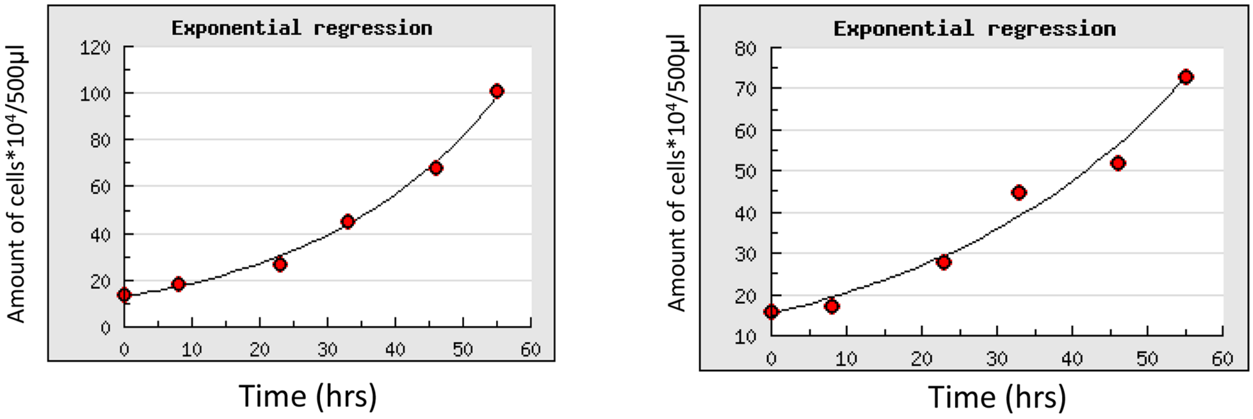

U20S = 18.8hrs

U20S Set7/9 knockdown = 24.5hrs

Supplementary Figure S1: Comparative growth curves of U2-OS control and Set7/9KD cells. (Upper table) Shown are the numbers of cells for each cell line after calculations taken after the indicated intervals of time. (Lower) Doubling time for U2-OS control and Set7/9KD cells calculated from the growth curves for each cell line.

A

Gene name	Set7/9KD 0 hrs	Set7/9KD 8 hrs	Set7/9KD 16 hrs	U2-OS 0 hrs	U2-OS 8 hrs	U2-OS 16 hrs	
Mdm2	1.2	4.2	3.2	1	3.0	3.1	p53-dependent genes
Cdkn1a	1.2	2.56	4.84	1	4.4	6.8	p53-dependent genes
Bax	-1.5	-0.1	1.0	1	1.15	2.5	p53-dependent genes
Tgfa	2.01	1.51	4.52	1	6.97	6.29	p53-dependent genes
Bbc3	-1.14	1.57	1.89	1	4.76	4.15	p53-dependent genes
Fas	2.02	2.3	5.3	1	5.6	7.2	p53-dependent genes
CCNE1	-1.4	-1.42	-1.73	1	1.05	1.17	p53-dependent genes
DDB2	-1.33	0.22	2.17	1	1.66	3.63	p53-dependent genes
Gadd45a	-1.39	0.83	1.26	1	2.64	2.28	p53-dependent genes
BTG2	-1.4	1.82	3.01	1	5.73	6.91	p53-dependent genes
PurB	-1.0	-1.16	-1.25	1	1.48	1.37	AR-dependent genes
Mre11A	1.5	1.14	1.06	1	-1.17	-1.32	AR-dependent genes
NBN	-1.62	-2.79	-2.63	1	-1.7	-1.72	AR-dependent genes
XRCC5	4.5	3.02	5.11	1	3.48	3.02	AR-dependent genes
RAD54B	1.12	-2.12	-2.1	1	-1.41	-1.18	AR-dependent genes
RAD51C	-1.72	0.38	1.47	1	1.82	3.41	AR-dependent genes
MSH6	-1.42	-1.76	-2.2	1	-1.17	-1.54	AR-dependent genes
Rad23B	-1.4	-0.72	-4.67	1	-1.31	-2.12	AR-dependent genes
TP73	3.01	1.02	3.78	1	1.0	-1.44	E2F1-dependent genes
UNG	-1.55	-0.33	-2.33	1	0.71	-1.58	E2F1-dependent genes
Prkdc	-1.48	-0.40	-1.1	1	-1.53	-2.53	E2F1-dependent genes
Pcna	-1.48	0.58	1.3	1	2.18	2.13	E2F1-dependent genes
Mad2L1	-1.9	-1.19	-3.26	1	-1.42	-1.46	E2F1-dependent genes
Fen1	-1.1	1.31	1.13	1	-1.0	-1.37	E2F1-dependent genes
TP53	-3.3	-1.21	-3.14	1	-1.22	-1.36	E2F1-dependent genes
Bub3	-1.76	-0.6	-2.33	1	-1.23	-1.1	E2F1-dependent genes

gene expression heatmap: -9-6 -6-4 -4-2 -2-0 0-2 2-4 4-6 6-8

B

Gene name	Set7/9KD 0 hrs	Set7/9KD 8 hrs	Set7/9KD 16 hrs	U2-OS 0 hrs	U2-OS 8 hrs	U2-OS 16 hrs
SetD7	-9.62	-8.62	-9.69	1	1.86	1.25
SetD5	2.83	0.82	2.08	1	-1.27	-1.53
KAT5	1.49	1.13	1.55	1	-1.51	-1.38
HDAC2	-1.5	-0.1	1.0	1	1.15	2.5

Supplementary Figure S2: Microarray gene expression analysis of U2-OS Set7/9 wild-type versus Set7/9 knock-down cells after treatment with doxorubicin. A. Genes that showed differential expression in U2-OS control and Set7/9KD cells in response to doxorubicin treatment for the indicated periods of time were selected according to their functions based on GO annotation (DNA repair and cell cycle) and then were clustered into three groups according to their master-regulators: p53, Androgen Receptor and E2F1. The gene expression heatmap keys are shown below. **B.** The gene list of histone modifying enzymes identified in the microarray gene expression experiment.

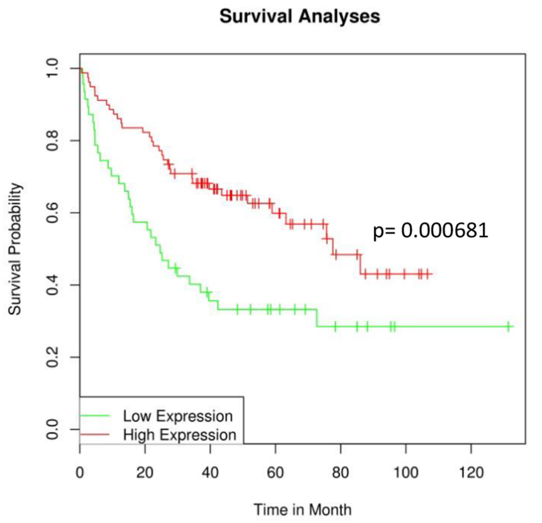

group=1: low expression of **SETD7**
group=2: high expression of **SETD7**

- GEO dataset ID: GSE39671
- GEO dataset Type: chronic lymphocytic leukemia

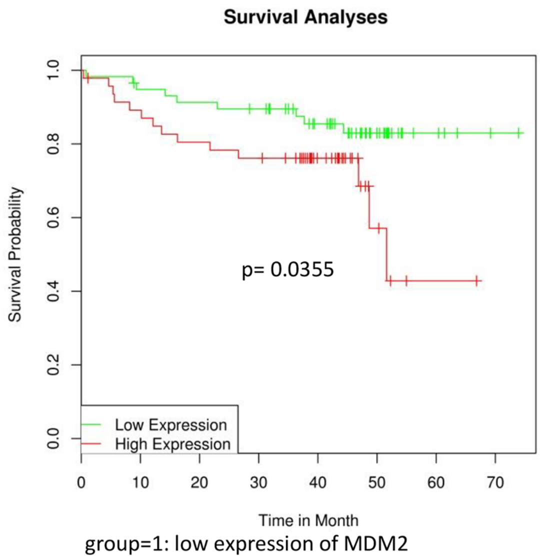

group=1: low expression of MDM2
group=2: high expression of MDM2

GEO dataset ID: GSE22762
GEO dataset Type: chronic lymphocytic leukemia

Supplementary Figure S3: Low expression of Mdm2 correlates with better survival of patients with chronic lymphocytic leukemia. The bioinformatics analysis demonstrates that high expression of Set7/9 **A.** and low expression of Mdm2 **B.** in chronic lymphocytic leukemia correlates with better survival.

gene	gene	correlation in p53 mut	correlation in p53 wt	p-value
SETD7	MDM2	**-0.17**	-0.01	**0.037**
SETD7	HDAC2	**0.03**	-0.02	**0.29**
SETD7	XRCC5	**0.15**	-0.00	**0.084**

Supplementary Figure S4: An illustration of the statistical procedure that divides samples into two groups based on the positive C. or negative correlation D. between Set7/9 and Mdm2. E. Effects of p53 status on correlations (shown in bold) between Set7/9 (SetD7) and Mdm2, or HDAC2, or XRCC5 expression levels were calculated using the METABRIC database. A statistically significant negative correlation between Set7/9 and Mdm2 was detected in breast cancer patients with mutated p53. *P*-values are shown for each case.
